# Performance Characterization of an Illumination-Based Low-Cost Multispectral Camera

**DOI:** 10.3390/s24165229

**Published:** 2024-08-13

**Authors:** Hedde van Hoorn, Angel Schraven, Hugo van Dam, Joshua Meijer, Roman Sillé, Arjan Lock, Steven van den Berg

**Affiliations:** 1 Photonics Research Group, The Hague University of Applied Sciences, 2628 AL Delft, The Netherlands; 2 Electrical Engineering, The Hague University of Applied Sciences, 2628 AL Delft, The Netherlands

**Keywords:** spectral imaging, low-cost, reflectance

## Abstract

Spectral imaging has many applications, from methane detection using satellites to disease detection on crops. However, spectral cameras remain a costly solution ranging from 10 thousand to 100 thousand euros for the hardware alone. Here, we present a low-cost multispectral camera (LC-MSC) with 64 LEDs in eight different colors and a monochrome camera with a hardware cost of 340 euros. Our prototype reproduces spectra accurately when compared to a reference spectrometer to within the spectral width of the LEDs used and the ±1σ variation over the surface of ceramic reference tiles. The mean absolute difference in reflectance is an overestimate of 0.03 for the LC-MSC as compared to a spectrometer, due to the spectral shape of the tiles. In environmental light levels of 0.5 W m^−2^ (bright artificial indoor lighting) our approach shows an increase in noise, but still faithfully reproduces discrete reflectance spectra over 400 nm–1000 nm. Our approach is limited in its application by LED bandwidth and availability of specific LED wavelengths. However, unlike with conventional spectral cameras, the pixel pitch of the camera itself is not limited, providing higher image resolution than typical high-end multi- and hyperspectral cameras. For sample conditions where LED illumination bands provide suitable spectral information, our LC-MSC is an interesting low-cost alternative approach to spectral imaging.

## 1. Introduction

In recent years, spectral imaging has become an integral part of the sensing toolkit in a number of fields. Applications range from the medical field [1], to remote sensing in earth observation [2], to agriculture and in particular precision farming [3]. Resolving more wavelengths than just Red, Green and Blue (RGB), as is common with both widely available cameras and our eyes, provides us with more detailed information about whatever it is we are looking at. As such, spectral imaging may help us to detect skin cancer [4], spot methane leaks [5] and provide us with a means to detect diseases on crops [6].

Typically, spectral imaging is divided into hyperspectral and multispectral imaging, denoting the difference between many spectral bands (in the order of 100) versus only several bands (in the order of 10), respectively [7]. This division has, however, become blurred and over-usage of the term *hyperspectral* is omnipresent in the literature [8]. In this research, we will still stick to the term multispectral camera as our approach is limited to nine bands (in this research we used eight of the nine available channels). Another important distinction in spectral imaging is the method (and thus speed) of acquisition [7], in particular snapshot versus scanning devices. Scanning can be performed inside the camera or by moving one spatial dimension of the sample. With our approach, we construct spectral information by sequential illumination with different wavelengths of interest, thus scanning along bands.

While a lot of information can be gathered from spectroscopy and spectral cameras, these devices remain very expensive. A short survey resulted in quotations of 6 to 12 thousand euros for multispectral (8–10 band) cameras, while a hyperspectral (about 100 bands) system costs 40 to 100 thousand euros (SWIR cameras being more expensive due to the InGaAs camera sensor). For certain applications such an investment may not be needed or affordable, and we therefore started investigating the possibility to develop a low-cost multispectral camera that may be applied to specific use cases instead of a costly high-end spectral camera.

In previous work, Bolton et al. developed a portable low-cost system costing about 1000 USD for medical applications, where they demonstrate the ability to measure a change in tissue composition [9]. With a similar LED-based Printed Circuit Board (PCB) approach, McCarthy et al. measured different samples to demonstrate the proof-of-principle that varying LED color illumination can yield spectral information of the object imaged [10]. Their device costs 885 euros and was used in an educational setting to demonstrate spectroscopy, chemometrics and colorimetry. Stuart et al. took a very different approach to make a low-cost hyperspectral imaging system using a self-made integrating sphere, while imaging with rotary mirrors scanning the light emitted from the object and while using a conventional grating-based spectrometer [11]. More recently, Orlando et al. demonstrated a low-cost imaging system with three visible and one infrared LED with an RGB camera without an IR filter, enclosed in a box to quantify vegetation indices of soil, turf and low vegetation [12]. All these approaches have up- and downsides, but have in common that a truly low-cost system can widen the use of spectral imaging in specific situations.

The ideal low-cost multispectral camera has multiple channels across the visible and near-infrared, is easy to handle and position in front of a sample and has low production costs that scale well with larger production numbers. In this paper we present a low-cost multispectral camera (LC-MSC), where we combine a low-cost system design (<500 euros, with potential for further cost reduction) with a quantification of its performance for reflectance measurement. Our system sequentially illuminates the sample with different color LEDs with wavelengths ranging from 400 nm to 1000 nm. At every illumination step we quantify the reflected intensity relative to the dark background using a monochrome camera without an IR filter. Relative to a spectrally flat, diffuse white reference under the same environmental conditions we then calculate the reflectance per pixel.

We demonstrate that our approach shows accurate spectral quantification when compared to a reference spectrometer on average within a mean absolute deviation in reflectance of 0.03. In the presence of bright artificial indoor illumination, we can still accurately measure the spectra of our reference tiles, though the reflectance variation does increase due to limited LED intensity for the lower and higher wavelength LEDs. In specific conditions where LED illumination bands provide suitable spectral information, this approach can be used as a low-cost alternative to expensive (hyper)spectral imaging.

## 2. Materials and Methods

Whereas most spectral cameras separate the wavelengths on the detector side (akin to a typical spectrometer), we set out to make a low-cost spectral camera that selects a sample wavelength response based on varying illumination. The basic principle is that by sequential illumination and camera acquisition with narrow wavelength bands, one can also reconstruct a spectrum at every spatial location in an image. When the sample does not move during illumination, a spectral hypercube can thus be reconstructed. In our design, there are nine channels available of each eight LEDs (we used eight channels and mounted 64 LEDs) with invisible and near-infrared (up to 1000 nm) wavelengths, fundamentally limited by the responsivity of our Si-based monochrome camera.

### 2.1. Prototype Design

We designed two Printed Circuit Boards (PCBs) to be used with a Raspberry Pi (RPi) 4. One PCB acts as a RPi HAT (Hardware Attached on Top), where voltage output and channel selection are regulated which is then connected to the second donut-shaped PCB where eight Light Emitting Diodes (LEDs) per channel are mounted with a current-regulating resistor. The total voltage drop over LEDs in series with a resistor can be set to 5 V, 12 V, 18 V, 24 V or 30 V. Voltage drop and resistor values are selected depending on the characteristics of the mounted LEDs (forward voltage and operating current). Power supply comes directly from the RPi with a DC–DC boost converter (Microchip MCP 1663). The LEDs can be dimmed by using Pulse Width Modulation (PWM) from the RPi at a frequency of 50 kHz, dimming intensity linearly at duty cycles of 5–95%. In the middle of the donut-shaped PCB with LEDs, a monochrome camera (Arducam OV2311) is mounted.

**Figure 1:**
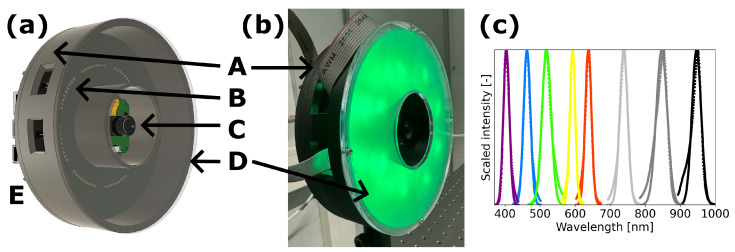
The low–cost multispectral camera (LC–MSC) prototype design and LED colors. In (**a**) schematic depiction and (**b**) photo of the realized prototype with green LEDs turned on. Denoted are (A) 3D printed PLA housing, (B) donut-shaped PCB with LEDs, (C) Arducam OV2311 monochrome camera, (D) diffuser and (E) Raspberry Pi 4 with PCB HAT (only slightly visible, mounted on the back). (**c**) shows normalized spectra of LEDs and Gaussian fits to determine spectral width of the 5 visible (plotted in their median color) and 3 infrared LEDs (in grey and black).

We made a holder from PLA (PolyLactic Acid) using a Prusa MK4 3D printer for the RPi, PCBs and camera. At a distance of 35 mm from the LEDs, we placed a diffuser (C-HH20-PE07-HE20, Brightview Technologies) to provide a field of illumination that is as homogeneous as possible, at sample location of 18 cm in front of the camera. Our prototype is controlled using Python code and a Graphical User Interface (GUI) written in PyQt5 where the RPi HAT is addressed using GPIO and the camera is read out through Arducam Pivariety control. The prototype design of our low-cost multispectral camera (LC-MSC), a photo of the realization and the LED spectra are shown in Figure 1.

### 2.2. Wavelength Selection and Costs

**Figure 2:**
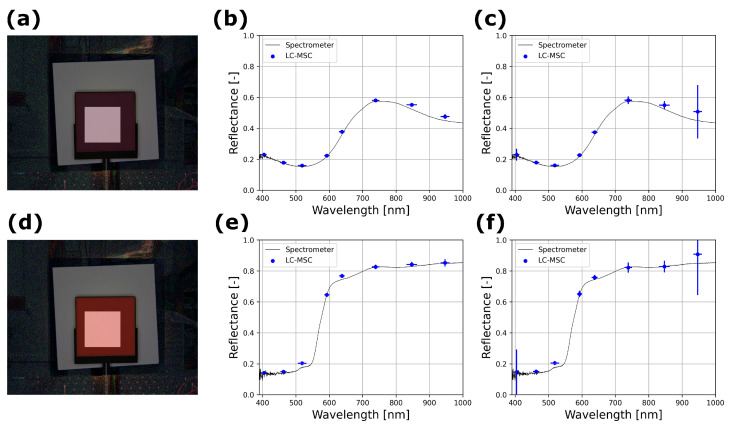
Spectral comparison for the Pink (top row) and Orange (bottom row) tiles (RGB-reconstructed images in (**a**,**d**)) show good correspondence to independently measured spectra in both dark (**b**,**e**) and indoor illumination (**c**,**f**) conditions. With indoor illumination conditions the variation in reflectance over the selected pixels (selection depicted as opaque white square) increases, in particular for the 405 nm and 950 nm LED, as their intensity is relatively low. Both multispectral images still show good correspondence to the (fully independent) reference measurement using a spectrometer, as given by the black line in (**b**,**c**,**e**,**f**).

**Figure 3:**
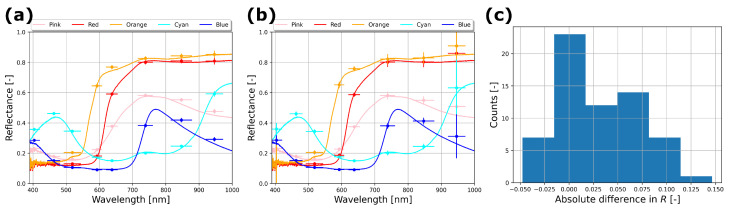
Spectral comparison of reflectance of various ceramic tiles without (**a**) and with (**b**) indoor illumination. Identical samples are color-coded and named according to the Ceramic Colour Standards Series II. Both the independent LC-MSC results (dots with error bars) and the spectrometer reference (full line in same color) are given. Error bar width is ±2δλ of a Gaussian fit to the LED spectrum and the reflectance uncertainty is given by ±1σ of the selected pixels on the tile. (**c**) histogram of absolute difference in *R* of the LC-MSC reflectance as compared to the spectrometer at the same narrow band shows a mean absolute difference of 0.03 over 8 colored reference tiles (more than shown in (**a**,**b**) alone).

The LC-MSC we present here has a broad wavelength range of LEDs over the full camera responsivity from 400 nm–1000 nm. We used Surface Mount Device (SMD) LEDs with center wavelengths at 950 nm, 830 nm, 740 nm, 630 nm, 589 nm, 525 nm, 465 nm and 405 nm [13,14,15,16,17,18,19,20]. Within the limited availability of low-cost LED colors, this provides broad spectral information so we can check whether we can quantitatively measure reflectance spectra. To quantify the spectral width we also measured the output spectra of the LEDs and fitted their scaled intensity to a Gaussian function (see Figure 1c). The reported spectral width in wavelength (error bars in Figure 2 and Figure 3) in our LC-MSC measurements δλ is the standard deviation of the Gaussian fitted to the LED spectra according to $I_{scaled}=e^{-{\left(\frac{\lambda -\lambda_{c}}{2\delta \lambda }\right)}^{2}}$.

The costs of our current prototype amounts to 140 euros for the monochrome camera, 80 euros for the RPi and 200 euros for PCBs, LEDs and other components. Note that when this approach would be scaled and further developed, a full RPi would no longer be needed and the PCB and camera costs would be significantly reduced. We believe that the material and fabrication costs could be reduced to below 200 euros. Current spectral cameras with wavelength selection on the detector typically cost about 10 thousand euros, where you also obtain 8–10 bands in the VISNIR range (with a Si-based detector).

### 2.3. Validation Measurements

To validate the spectral imaging capability of our LC-MSC, we measured the spectra of matt Ceramic Colour Standards tiles—Series II (CCSII, Lucideon). We quantified reflectance *R* over a wavelength range of 390–1000 nm relative to a 95% diffuse reflectance reference (Zenith SG 3151, Sphere Optics). With R=0.95 for the white reference and compensating for dark signal at every wavelength, we calculated *R* at different wavelengths according to

(1) $$R=\frac{I_{\mathrm{sample}}-I_{\mathrm{dark}\;\mathrm{sample}}}{I_{\mathrm{reference}}-I_{\mathrm{dark}\;\mathrm{reference}}}.$$

$I_{\mathrm{sample}}$ refers to the irradiance at the pixels when the sample is illuminated, $I_{\mathrm{dark}\;\mathrm{sample}}$ is the irradiance while the LED illumination on the sample is turned off but environmental light levels are present. $I_{\mathrm{reference}}$ gives the reference pixel values when the white reference is imaged with LED illumination and $I_{\mathrm{dark}\;\mathrm{reference}}$ means the white reference is still in place but LED illumination is turned off. For all irradiance levels, the exposure time, camera gain and environmental conditions are kept constant. RGB images are reconstructed by displaying the reflectance values *R* for each pixel (no further image processing is employed) with Red, Green and Blue LED illumination, as depicted in Figure 2a,d.

Next to measurements using our LC-MSC, we quantified the reflection spectra using an Avantes AvaSpec-ULS2048CL-EVO spectrometer with a 25 μm slit, coupled with a FC-UVIR200-2 optical fiber to an integrating sphere. We illuminated the samples with the integrated broadband halogen light source in the integrating sphere, Avantes AvaSphere-50-LS-HAL-12V. We also measured spectra with the LC-MSC with varying background illumination, applied with a broadband white LED source. We used an irradiance level of up to 0.5 W m^−2^ background illumination on the location of the sample, corresponding to bright indoor illumination. Our LC-MSC irradiance at the center of the sample supplied by the LC-MSC varied between 3 and 80 W m^−2^ (depending on the LED) when the object was placed at 18 cm from our prototype. With a sample placed at this distance, the entire white reference was in our field of view and the illumination remains bright relative to normal indoor lighting conditions. We measured spectra using the LC-MSC with a set exposure time while varying the illumination intensity, as well as using maximum illumination using varying exposure times. Both approaches yielded similar spectral correspondence between the reference spectra measured by the spectrometer and the LC-MSC.

## 3. Results and Discussion

Using our prototype spectral camera (see Figure 1) we acquired a multispectral image by sequentially turning on eight different color LEDs. We tuned the camera such that it gives a linear response to irradiance by disabling automated correction. Images are always acquired relative to dark and white reference after which reflectance is computed according to Equation (1). When the exposure time is kept constant, only two dark references need to be taken (with white reference and with sample), otherwise with varying exposure times a dark reference is taken for each exposure time. Since the camera needs to stabilize its response for approximately 1 s after changing exposure time, keeping the exposure time constant and varying illumination intensity greatly speeds up the total acquisition of one multispectral image. With varying exposure time the acquisition of a single frame takes up to approximately 10 s, whereas with constant exposure time per LED this speeds up to less than 1 s.

Figure 2 shows images and spectra quantified with our LC-MSC compared to fully independent reference measurements using the spectrometer with a broadband light source. The opaque white mask depicted in (a) and (d) shows the pixels over which the LC-MSC spectra were computed in (b,c) and (e,f). The tiles are measured in either dark conditions (b,e) or with all indoor lighting (c,f) in the lab, including illumination from right above the optical table producing a background irradiance on the tile of 0.5 W m^−2^. When the spectra are compared, they show good correspondence, especially where the spectral reference curve from the spectrometer is fairly flat or there is a monotonous increase or decrease in the spectrum. When there is a sudden increase or decrease in reflectance at the edge of the LED spectrum, as with the 525 nm LED in e-f, we observe a deviation in reflectance. This can be explained by the spectral bandwidth of the LEDs, as the 525 nm LED has an emission tail to higher wavelengths where the reflectance of the tile (or in general, a sample) is higher so the overall reflectance at 525 nm is overestimated by the LC-MSC.

Figure 3 gives a quantitative comparison of reflectance spectra for multiple tiles. This figure shows that the overall shape of spectra over the full range of low-to-high reflectance from 400 to 1000 nm is faithfully reproduced by the LC-MSC, as compared to the fully independent spectrometer measurement. The mean absolute deviation in *R* is 0.03 relative to the spectrometer reflectance (for eight different tiles over all eight LED colors, see Figure 3c), where the LC-MSC mostly overestimates the reflectance. The eight tiles used had varying spectra, while the other four CCSII tiles were different grayscales and showed good correspondence as well. Only five of the eight varying spectra are plotted for clarity in Figure 3. This overestimation in *R* is because there is often a reflectance increase spectrally adjacent to the LED mean where there is still a spectral tail of LED illumination, as visible in several of the curves in Figure 3a,b. We observe that at the low and high wavelengths, at the 405 nm and 950 nm LEDs, the variation of the observed reflection increases. This can be explained by the fact that both LEDs have a relatively low intensity compared to the other LEDs. Keep in mind that our camera always observes all wavelengths, so with background illumination we have a significant dark offset. Still, the reflectance, even with indoor illumination as shown in Figure 3b, corresponds well to the spectrometer results.

With very high intensity background illumination our approach does reach a fundamental limitation, as the bit depth that is left over on the camera for reflectance quantification becomes too small. The threshold background illumination is dictated by the lowest irradiance illumination intensity by a LED. In our configuration the background irradiance threshold is 2.6 W m^−2^ (this is the illumination irradiance of the 405 nm UV LED), which is still 5× higher than the total irradiance of artificial indoor lighting in the vertical plane. Also, we need the background illumination to be constant during our sequential illumination, so we can subtract a constant dark background. Furthermore, to keep this a low-cost approach we must use spectrally separate LEDs that are available at low cost, which limits the wavelength bands that can be used. And of course, the spectral width in the order of 10s of nm for LEDs limits the spectral resolution. So if you need to probe either a very specific narrow band or need very high spectral resolution, our low-cost technique may not be suitable. However, unlike with many conventional spectral cameras, the pixel pitch of the camera itself is not limited in this approach. This means that we have an added benefit (beside the low cost), which is that we can provide a higher image resolution than typical high-end multi- and hyperspectral cameras.

## 4. Conclusions

In this article, we have demonstrated the design and realization of a low-cost multispectral camera (LC-MSC). With PCBs, LEDs, a monochrome camera and a Raspberry Pi we designed and realized our prototype. Both in dark and standard indoor illumination conditions, we can image and faithfully reproduce reflectance spectra. Due to the spectral bandwidth of the LEDs we cannot reproduce narrow spectral features and the mean wavelength may over- or underestimate the reflectance when steep curves occur in the spectra. When we turn on indoor illumination, this increases the uncertainty of the acquired spectra, especially at the 405 nm and 950 nm LED. The mean absolute difference in reflectance is 0.03, where the LC-MSC typically overestimates the reflectance due to the spectral shape of the reference tiles. We also observe some limitations on the availability of LEDs at specific wavelengths to the applicability of this proposed approach. Overall, for specific applications, our LC-MSC may be an excellent low-cost alternative to expensive spectral imaging.

## Data Availability

The data presented in this study are openly available in a data repository via https://hhs.data.surfsara.nl/index.php/s/iMzQ8GKWgNmXUl3 (accessed on 12 July 2024), password: THUASPho24!.
